# Hostile and energetic: Anger is predicted by low agreeableness and high energetic arousal

**DOI:** 10.1371/journal.pone.0184919

**Published:** 2017-09-20

**Authors:** Marcin Zajenkowski

**Affiliations:** Faculty of Psychology, University of Warsaw, Warsaw, Poland; Boston Children's Hospital / Harvard Medical School, UNITED STATES

## Abstract

A hypothesis that anger is related to high energetic arousal (EA), but only at a low level of agreeableness was tested. In the first two studies, the association between trait anger, agreeableness, and EA was explored. In the next two studies, the interactions of agreeableness with constructs conceptually and empirically close to EA, in predicting anger were tested. Study 3 revealed that anger was predicted by a low level of agreeableness and a high level of positive affect. In study 4 task engagement (construct containing an energetic component) was included in the context of cognitive performance. At a high level of agreeableness the association between anger and task engagement was negative, whereas among individuals with low agreeableness this relation was not significant.

## Introduction

Frequent and intense experiences of anger have a number of adverse consequences including health problems [1], an increased likelihood of aggression [2], and relationship difficulties [3]. Thus, understanding the nature of anger is of high social importance. Recently, a growing interest in examining the structure of state and trait anger is observed. One example is a work of Bresin, Hilmert, Wilkowski, and Robinson [4], who referred to the two-dimensional model of emotional experience according to which emotion is a combination of valence (negative versus positive) and arousal (high versus low; [5]). Bresin et al. [4] pointed to the fact that, on the one hand high arousal conditions often increase the intensity of aggressive feelings and responses [6], while on the other hand, anger is usually accompanied by hostile thoughts (e.g., [7]). Therefore anger (state and trait) might be seen as an interactive combination of hostile attitude and high arousal [4]. Bresin et al. [4] identified the former with low agreeableness and the latter with high response speed. The authors referred to the data showing that disagreeable individuals have hostile thoughts, are cynical concerning others, and are cold in their interpersonal functioning [1], [8]. Additionally, they reviewed psychological literature reporting that arousal is positively associated with response speed (e.g., [9]). In a series of studies Bresin et al. [4] confirmed their hypothesis, that anger is best predicted by an interaction of low agreeableness and high response speed. What might be problematic, however, is the use of indirect measure of arousal. Although there is some evidence that arousal is associated with faster reactions (e.g., [9]), it would be valuable to show the link between anger and more direct instruments measuring arousal. Additionally, the widely accepted view put forward by Thayer [10] is that arousal might be a complex and multidimensional construct. Specifically, Thayer [10] distinguished between two subjective states: energetic arousal (EA, or feelings of energy vs. fatigue) and tense arousal (TA, tension vs. calmness), linking the former with mobilization and approach motivation. In fact, Bresin et al. [4] refer also to Thayer's concept of EA considering response speed as an indicator of arousal.

Other investigations that may shed light on the structure of anger have been conducted by Harmon-Jones and colleagues [11], [12], [13], who argued that anger is related to approach behavior, and because of that, consists of both negative affect (NA) and positive affect (PA). The somewhat counter-intuitive conclusion related to PA has been based on experimental and correlational evidence. Harmon-Jones et al. [12], in a series of experiments, tested whether PA measured by the Positive and Negative Affect Schedule (PANAS; [14]) was associated with state anger. The authors observed that anger evoking situations produced higher levels of anger and PA than neutral conditions without emotion induction. Moreover, they found that PA was positively correlated with anger, anger and happiness were inversely correlated, and happiness and PA were positively correlated. Statistically controlling for happiness increased the size of the correlation between PA and anger. Harmon-Jones et al. [12] concluded that PA includes two aspects, i.e., positive emotionality and approach motivation, and its positive association with anger is due to the latter one. Statistically controlling for happiness removes the positivity of PA and increases the magnitude of the PA-anger relationship. Thus, anger might be described as an approach-oriented, but negatively valenced emotion. In a follow-up study, Harmon-Jones and Harmon-Jones [13] have shown that happiness also acts as a suppressor when trait anger and trait PA are analyzed. Specifically, the relationship between PA and anger becomes statistically significant when happiness is controlled in the model.

Harmon-Jones and colleagues [12] [13] conclude that the use of factor analysis in the development of the PA (selection of items with a large loading on the one factor and a near-zero loading on the other factor), resulted in elimination of items that measure pure positivity and retention of items that measure additional aspects, such as approach motivation. Harmon-Jones et al. [12] emphasize that their findings are consistent with the PANAS theory, because Watson [15] linked PA with approach behavior and claimed that it might be a part of a larger Behavioral Activation System (BAS). The latter has been shown to correlate with both state and trait anger [11].

### Current studies

The purpose of the present investigation was to advance the research on trait anger by revealing its specific aspects. Building on previous data linking anger with approach and arousal [4], [13], I aimed to examine whether trait anger might be described as a mixture of high arousal/approach and negative/hostile attitude. Something that should be considered theoretically is the relationship between approach motivation and arousal. In the model proposed by Thayer [10], to which I will refer in the present research, energetic arousal is an indicator of mobilization and *fight* behavior and thus represents approach motivation. Harmon-Jones et al. [12], however, suggest that approach motivation might be distinguished from arousal. In one of their studies showing that PA is linked to anger, they included additional items referring to arousal: *aroused* and *stimulated*. Harmon-Jones et al. [12] concluded that anger was positively linked with PA even after controlling for the arousal words. However, the approach-arousal relation might be more complicated. Interestingly, some researchers point out the conceptual similarity between PA and EA [16], and the careful analysis of the tools assessing both constructs reveals that indeed they may refer to the same phenomenon. Specifically, some of the items in the EA scale overlap with the PA scale (e.g. *active*, *alert*), while others capture similar states of being active and approach-oriented (*energetic*, *passive*, *vigorous* in EA; *enthusiastic*, *excited*, *strong* in PA). Although attempts have been made to show differences between motivation and arousal in emotional experience [17], it is particularly difficult to distinguish clearly EA from PA, at least at the self-report level of measurement. Rather, both EA and PA, refer to a broader state of high approach motivation, activation and energy.

In the present investigation, four independent studies were conducted showing that anger is associated with high energy (and related constructs), but only at low level of agreeableness. In the first two studies the association between trait anger, trait agreeableness and arousal understood as a mood dimension [10], [17] was explored. Unlike prior investigations [4], a more direct index of arousal was included. Specifically, I referred to Thayer’s [10] suggestion that among the two arousals, it is EA that is associated with mobilization of energy observed in anger episodes. In the next two studies, I wanted to examine whether agreeableness interacts with constructs related to EA, containing also the energetic and approach components, in predicting anger. In study 3, the interaction between agreeableness and positive affect measured with the PANAS was tested. As was noticed above, PA might be conceptually close to EA, and it is difficult to clearly conclude whether PA captures motivation or arousal. In study 4, task engagement, a construct developed by Matthews et al. [18] was included in the context of cognitive performance. Task engagement includes EA as one of its core components. In all cases, it was expected that anger would be predicted by a low level of agreeableness and a high level of EA related variables.

## Method

### Ethics statement

The studies were approved by the ethics committee of Faculty of Psychology at University of Warsaw. Verbal informed consent was obtained from all participants after careful information about the general aim of each study, the study procedure and protocol clearly mentioning the possibility to withdraw from participating in the study. The studies entailed healthy, adult individuals and the procedures did not infringe on the integrity of the subjects, did not include any experimental manipulation, any sample taking or administration of any substances but rather were based on standard tests and questionnaires. In this situation written consent was not required by the ethics committee.

All materials, including questionnaires and data were uploaded to Open Science Framework and are available under the following address: osf.io/43pga.

### Study 1

Study 1 constituted an initial examination of the hypothesis that trait anger will be predicted by the interaction of low agreeableness and high energetic arousal. Energetic arousal is usually measured as a temporary state and that is how it was assessed in study 1.

#### Participants

A total of 380 participants took part in the study (274 female and 106 male). Their mean age was 21.50 (SD = 3.60) with a range of 18 to 47. The sample was composed of undergraduate students from the University of Warsaw and the Academy of Special Education in Warsaw, Poland. Participants were tested in groups of 30–50 people at the beginning of a lecture. Eight of the approached participants declined to take part in the research. The study was conducted between May and June 2014.

#### Materials

Energetic arousal was assessed with a Polish adaptation (cf. [19]) of the University of Wales Mood Adjective Check List (UMACL; [17]). This is a self-report scale which includes 10 items measuring EA (with poles: energetic–tired). In the testing procedure participants are asked to rate themselves on a scale, ranging from 1 (*strongly disagree*) to 4 (*strongly agree*). In the present study the mean was 28.46 (SD = 5.61) and α = 0.84.

Agreeableness. The NEO Five Factor Inventory (NEO-FFI) [20] in the Polish adaptation [21] was used to measure agreeableness. This scale contains twelve items with a 5-point Likert-type scale. In the present sample the mean was 28.83 (SD = 6.25) and α = 0.72.

Trait anger was assessed with the Aggression Questionnaire (AQ; [22]). It contains seven items (e.g. “Some of my friends think I’m a hothead”) and uses a 5-point Likert-type scale to score the items. In the present sample the mean was 19.92 (SD = 5.30) and α = 0.70.

#### Results

All data from studies 1–4 were uploaded to Open Science Framework and are available under the following address: osf.io/43pga.

**Fig 1:**
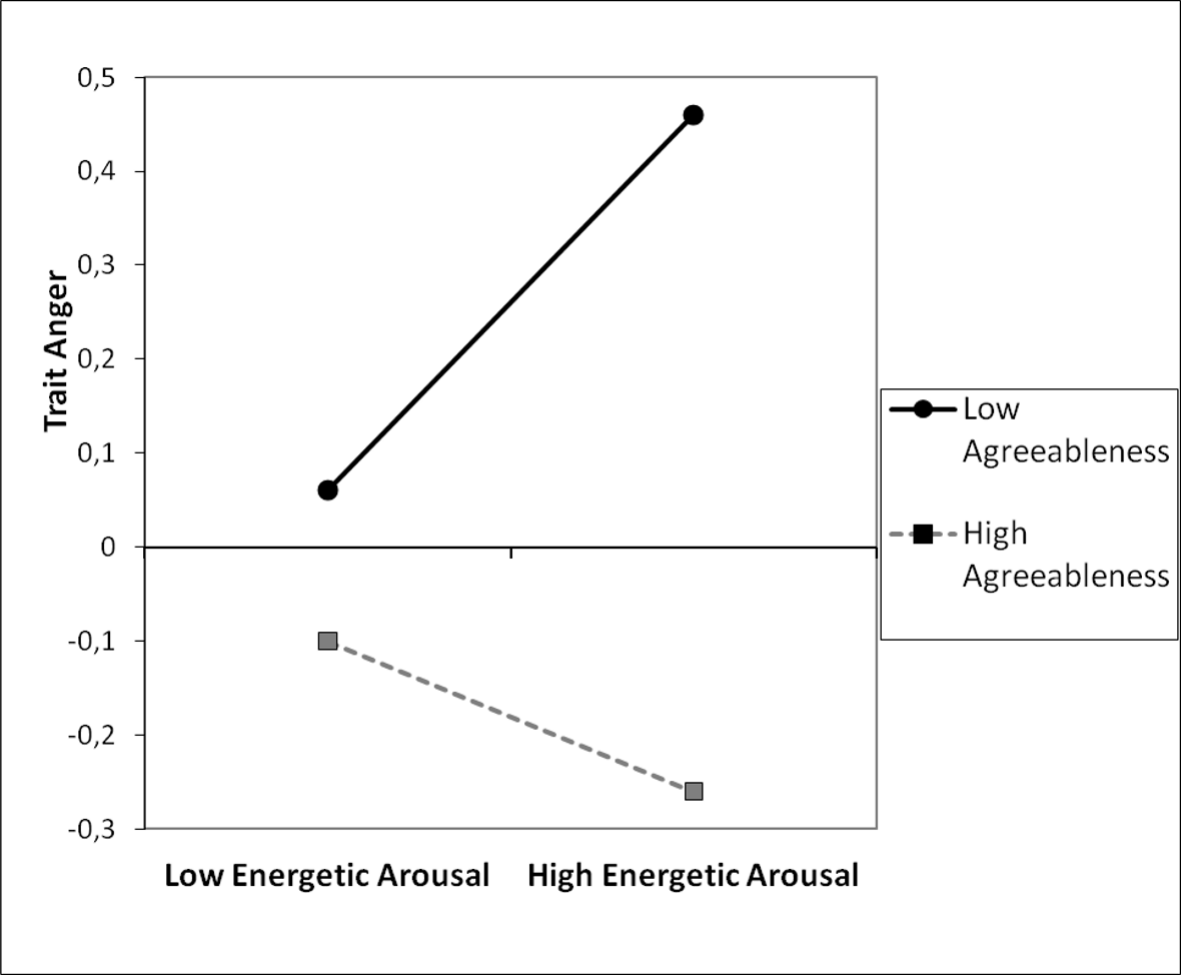
Trait anger as a function of agreeableness and energetic arousal, study 1.

Correlations revealed that anger was negatively related to agreeableness (r = -.21; p < 0.001), agreeableness was positively correlated with EA (r = .25; p < 0.001), finally, anger and EA were not significantly associated (r = -.02; p = 0.752). To test whether agreeableness and EA interact in predicting anger a regression model with agreeableness, EA, and an interaction term (agreeableness x EA) as predictors and anger as the dependent variable was run. The variables were standardized. The model was significant (F(3, 376) = 8.88; p < 0.001) and accounted for 7% of the variance. The interaction was significant (see Table 1). To decompose the interaction, we examined the simple slope of energetic arousal among individuals high in agreeableness (1 *SD* above the mean) and low in agreeableness (1 *SD* below the mean). Fig 1 shows graphically that at low level of agreeableness, the association between EA and anger was more pronounced and positive (β = 0.20, *t* (376) = 2.57, *p* = .011), while for individuals high in agreeableness this relationship was not significant (β = -0.08, *t* (376) = -0.84, *p* = .230). The Johnson-Neyman regions of significance test revealed that the effect of EA was significant and positive among individuals who scored -0.40 of a standard deviation or lower on agreeableness; whereas among participants who scored 1.84 of a standard deviation or higher on agreeableness, the effect became negative.

**Table 1:** Regression model with agreeableness, energetic arousal, and agreeableness x energetic arousal interaction as predictors and anger as a dependent variable.

	β	p
Agree	-.22	.000
EA	.06	.263
Agreeableness x EA	-.14	.006

*Note* EA–energetic arousal, Agree—Agreeableness

#### Discussion

Prior studies have shown that anger is related to approach motivation [12], hostile attitude [7] and disagreeableness [4]. Building on these findings, I hypothesized that anger will be predicted by a low level of agreeableness and high energetic arousal. Study 1 confirmed this hypothesis. It is important to note that a direct measure of energetic arousal was used, while in prior work [4] the interaction was tested with response speed as an indicator of arousal. What might limit the conclusions is the fact that only momentary state of EA was assessed, while trait anger is a general tendency for certain reactions. Therefore, in a follow-up study, I decided to address this problem.

### Study 2

EA is a temporary state and that is how it was assessed in study 1. To determine whether anger is associated with high EA over longer period of time, in study 2, EA was assessed twice with a one-week gap between the measurements. As in study 1, a statistically significant interaction between the mean EA from the two assessments and agreeableness in predicting trait anger was expected.

#### Participants and procedure

A total of 123 participants took part in the study (103 female and 20 male). Their mean age was 20.22 (SD = 1.80) with a range of 18 to 31. The sample was composed of undergraduate students from the Academy of Special Education in Warsaw, Poland. Participants were tested with exactly the same measures as in study 1, however, in study 2, energetic arousal was assessed twice at the same lecture with a one-week break between measurement sessions. The study was conducted in April 2014. One hundred fifty participants were initially approached and 9 of them refused to participate in the study. The drop-out rate was approximately 12% (participants taking part only in one session).

#### Results

Paired sample t-test revealed no differences between first and second measurements of EA (t(122) = 0.76; p = .450). Thus, I decided to calculate the mean score of the two EA measurements for each participant. Next, all the variables were correlated with one another (see Table 2). The analysis indicated that agreeableness correlated negatively with anger, and positively with EA (first measurement and the mean score), whereas anger did not exhibit significant associations with EA. Finally, there was a positive association between energetic arousal as measured on two occasions. The latter relationship was moderate which is typical for mood scales assessing momentary state [16].

**Table 2:** Correlations between anger, agreeableness, and energetic arousal.

	Anger	Agreeableness	EA 1	EA 2	EA mean
Anger	-	-.45**	.02	-.03	-.01
Agreeableness			.18*	.14	.19*
EA 1				.46**	.86**
EA 2					.85**
M (SD)	19.11 (5.30)	30.81 (7.00)	29.79 (5.65)	29.40 (5.52)	29.59 (4.77)
α	.72	.80	.83	.87	-

*p < 0.05

**p < 0.01

*Note* EA–energetic arousal

**Fig 2:**
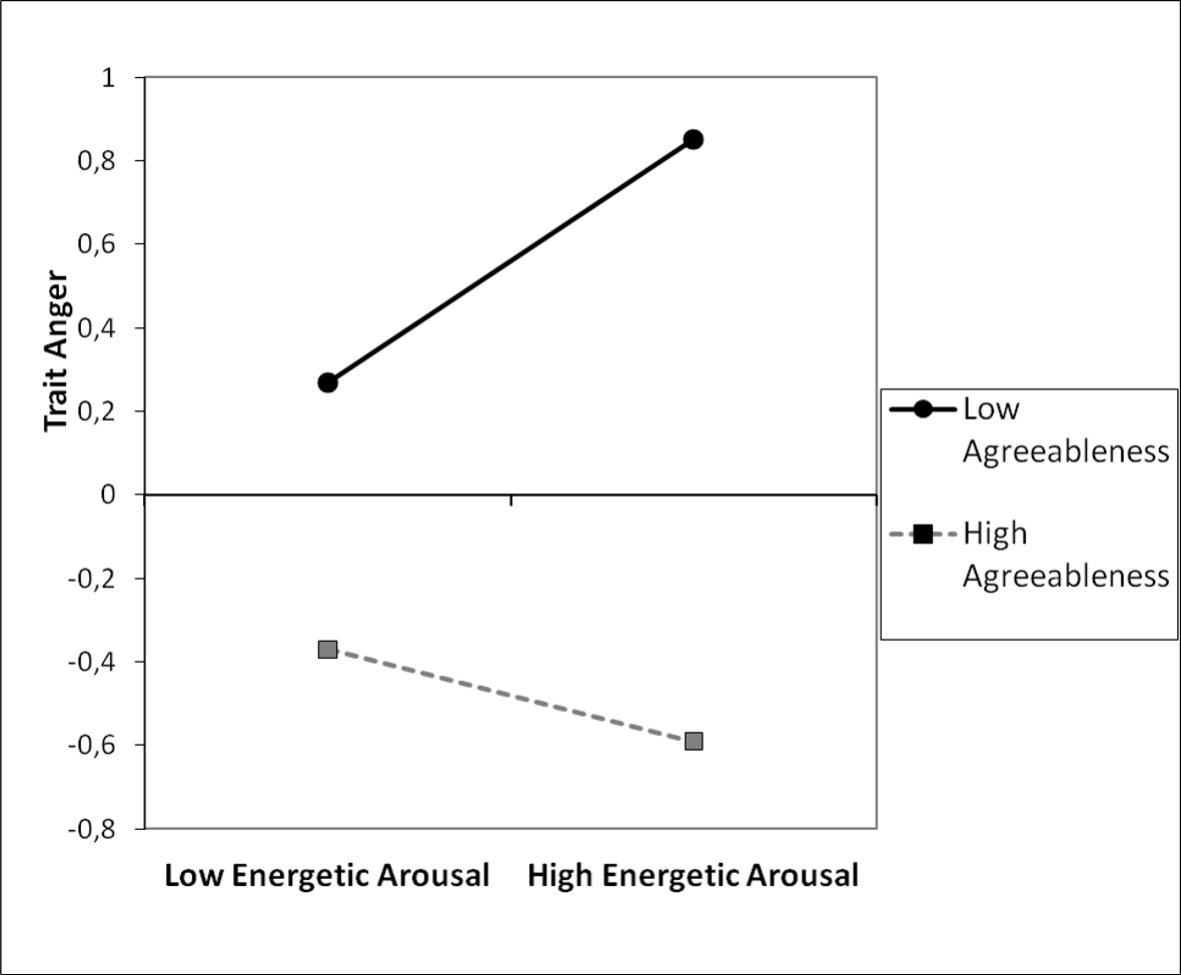
Trait anger as a function of agreeableness and energetic arousal (the mean score from two measurements), study 2.

Next, regression analyses were conducted with agreeableness, EA, and an interaction term (agreeableness x mean EA) as predictors and anger as the dependent variable (see Table 3). The interaction was significant for the mean EA. Subsequently, I examined the simple slope of energetic arousal (the mean score) among individuals high in agreeableness (1 *SD* above the mean) and low in agreeableness (1 *SD* below the mean). Fig 2 shows graphically that at low level of agreeableness, the association between EA and anger was more pronounced and positive (β = 0.28, *t* (119) = 2.40, *p* = .018), while for individuals high in agreeableness this relationship was not significant (β = -0.10, *t* (119) = -0.91, *p* = .364). The Johnson-Neyman regions of significance test revealed that the effect of EA was significant and positive among individuals who scored -0.46 of a standard deviation or lower on agreeableness.

**Table 3:** Regression models with agreeableness, energetic arousal, and agreeableness x energetic arousal interaction as predictors and anger as a dependent variable.

	β	p
Agree	-.52	.000
EA mean	.09	.260
Agree x EA mean	-.20	.021

*Note* EA–energetic arousal, Agree—Agreeableness

#### Discussion

The results obtained in study 2 further supported the hypothesis that trait anger is described as a combination of disagreeableness and high energy. This time, EA was assessed twice with a one-week gap between measurements. The results indicated that anger was associated with a high mean level of EA, but only when coupled with low agreeableness. It need to be acknowledged, however, that the short gap between the two EA measurements may be a potential limitation of the aforementioned conclusion. Empirical research show that personality traits are relatively stable over longer periods of time, i.e. years or even decades [16]. Thus, EA measured on two occasions with a one-week break between sessions does not provide information about trait-like assessment of energy.

Studies 1 and 2 confirmed our basic hypothesis. However, it would be interesting to see whether other constructs conceptually and empirically close to EA reveal similar effects. Two of them seem especially interesting. First, it has been suggested that positive affect might be associated with anger [12], because it contains an approach aspect. Thayer [10] emphasized that EA is also associated with *fight* rather than *flight* behavior. Paradoxically, PA might be related to anger in a similar way to EA–through low agreeableness. Second, it would be valuable to examine the association between anger and energy in a context that is more significant for real life such as, for instance, high effort in cognitive performance.

### Study 3

It has been shown that PA taps positivity as well as approach motivation, and this approach-motivated affect can occur in anger evoking situations. Interestingly, researchers point out the conceptual similarity between PA and EA [17], and the careful analysis of the tools assessing both constructs reveals that indeed they may refer to the same phenomenon. Therefore, in study 3, we tested whether agreeableness interacts with PA in predicting anger. We expected a positive correlation between trait anger and PA at the low level of agreeableness, similar to our results on EA from studies 1 and 2.

#### Participants

A total of 176 participants took part in the study (99 female and 81 male). Their mean age was 23.90 (SD = 4.13) with a range of 18 to 39. The sample was composed of undergraduate students from various universities in Warsaw, Poland. Participants were recruited via social media. No one refused to participate in the study. The study was conducted between March and June 2015.

#### Materials

Trait anger was measured with the same scale as in study 1. In the present sample the mean was 18.53 (SD = 5.92) and α = 0.83.

Agreeableness was measured with the Polish adaptation [23] of the 50-item set of International Personality Items Pool Big Five Factor Markers questionnaire [24]. The agreeableness scale consists of 10 items with a 5-point Likert-type response format, from 1 –very inaccurate to 5 –very accurate. The reliability and validity of the Polish version was tested on a large sample, showing high internal consistency (α = 0.81; [24]). In the present sample the mean was 39.15 (SD = 5.40) and α = 0.78.

Positive affect was assessed with the PANAS PA subscale [14] in the Polish adaptation by Brzozowski [25]. It asks participants to rate their momentary affective experience and consists of ten items with a 5-point Likert-type response format, from 1– very slightly or not at all, to 5 –extremely. In the present sample the mean was 30.34 (SD = 6.42) and α = 0.86.

#### Results

**Fig 3:**
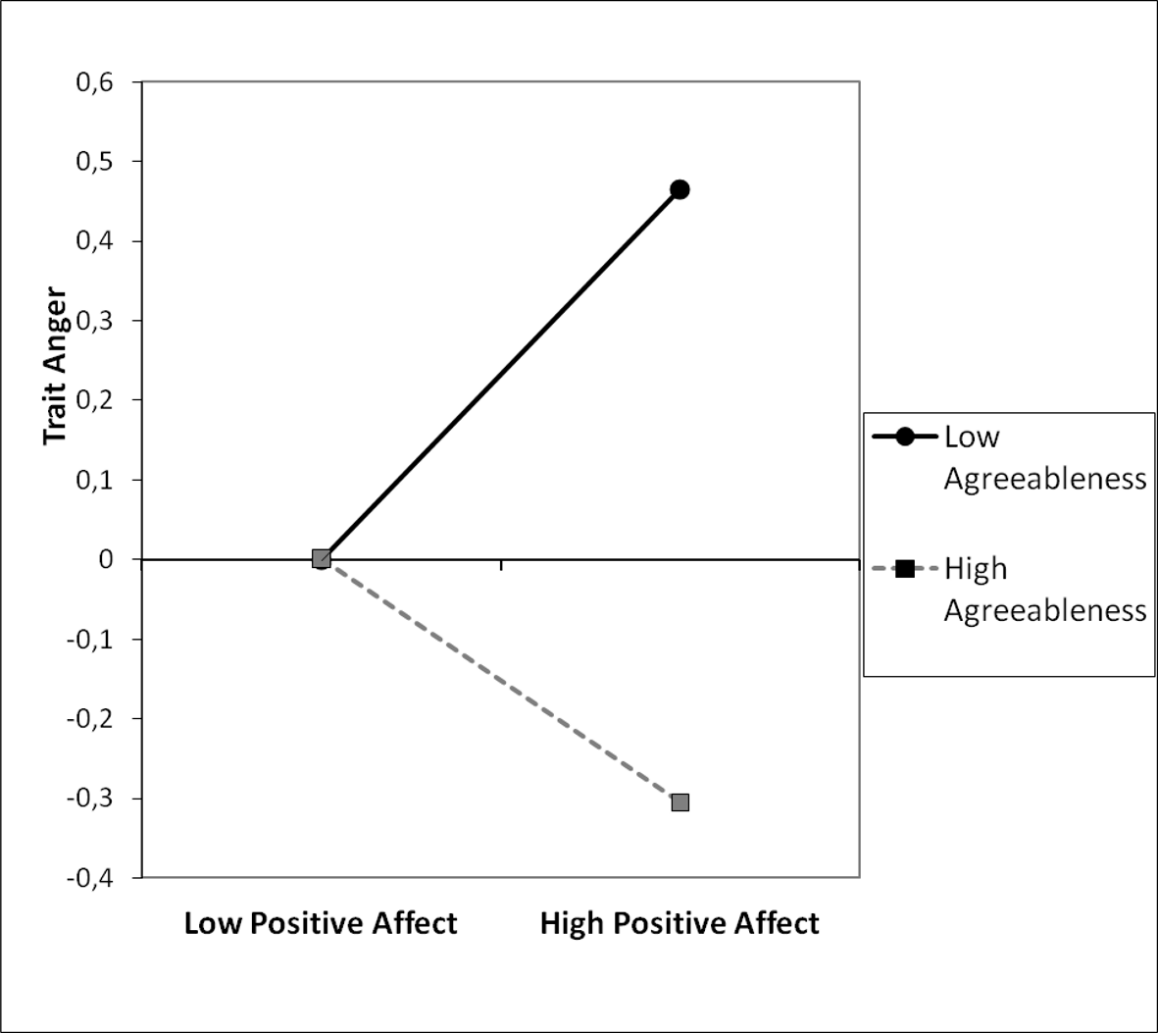
Trait anger as a function of agreeableness and positive affect, study 3.

The correlations among variables indicated that anger was negatively related to agreeableness (r = -.20; p = 0.008), agreeableness was positively correlated with PA (r = .19; p = 0.012), finally, anger and PA were not significantly associated (r = -.02; p = 0.773). Further, we tested whether agreeableness and PA interact in predicting anger. A regression model with agreeableness, PA and an interaction term (agreeableness x PA) as predictors and anger as the dependent variable was run (Table 4). The variables were standardized. The model was significant (F(3, 175) = 4.53; p = 0.004) and accounted for 7% of the variance. The interaction was significant (see Table 4). Subsequently, we examined the simple slope of positive affect among individuals high in agreeableness (1 *SD* above the mean) and low in agreeableness (1 *SD* below the mean). Fig 3 shows graphically that at low level of agreeableness, the association between PA and anger was more pronounced and positive (β = 0.23, *t* (172) = 2.13, *p* = .034), while for individuals high in agreeableness this relationship was not significant (β = -0.16, *t* (172) = -1.58, *p* = .117). The Johnson-Neyman regions of significance test revealed that PA was positively associated with anger among individuals who scored -0.86 of a standard deviation or lower on agreeableness; whereas among participants who scored 1.46 of a standard deviation or higher on agreeableness, the effect was negative.

**Table 4:** Regression model with agreeableness, positive affect, and agreeableness x positive affect interaction as predictors and anger as a dependent variable.

	β	p
Agreeableness	-.19	.011
PA	.04	.625
Agreeableness x PA	-.19	.009

*Note* PA–positive affect

#### Discussion

Past studies indicated that trait anger is directly correlated with PA when statistical controls for positive valence are implemented, which suggests that it is the approach motivational part of PA that correlates with anger [24]. The current study extends these findings by showing that trait anger is positively associated with PA at the low level of agreeableness. This result is consistent with our studies 1 and 2, and we believe it is because PA is highly loaded by energetic and approach aspects. Importantly, Harmon-Jones et al. [12] asked participants about their attitude toward anger, and found that anger was evaluated as a negative experience. The authors concluded that anger is similar to certain positive affects not due to anger being felt as positive, but rather that anger and PA are associated with approach motivation.

### Study 4

In the last study we wanted to examine to what extent the previously observed effects generalize to other constructs related to EA. This time we used task engagement as an indicator of approach motivation and energy. Task engagement is a part of a multi-dimensional model of subjective stress state related to cognitive performance proposed by Matthews et al. [18]. The authors identified three broad factors: task engagement, distress, and worry. However, in the present research we were interested only in the former. Task engagement integrates state constructs that relate to task interest and focus: energetic arousal, motivation, and concentration. Thus, it directly includes aspects of an approach state (motivation, energy). In the current research, we assessed task engagement in the context of solving a demanding cognitive task, i.e., a fluid intelligence test. It has been shown that stress states may explain the associations between some personality traits (e.g., trait anger) and the score on an IQ test [26], [27].

#### Participants

A total of 189 participants took part in the study (101 female and 88 male). Their mean age was 22.30 (SD = 2.69) with a range of 18 to 34. The sample was composed of undergraduate students from various universities in Warsaw, Poland. Participants were recruited via social media. The study was conducted between November 2014 and January 2015. One hundred ninety three participants were initially approached and 4 of them refused to participate in the study.

#### Materials

Information about reliability and descriptive statistics of all measures are presented in Table 5.

**Table 5:** Correlations between anger, agreeableness, task engagement, and Raven’s.

	Anger	Agreeableness	Task Engagement pre-task	Task Engagement post-task	Raven
Anger		-.21**	-.14	-.15*	-.16*
Agreeableness			.23**	.08	-.07
Task Engagement pre- task				.49**	-.02
Task Engagement post- task					.31**
M (SD)	21.81 (4.83)	37.90 (6.10)	20.92 (4.30)	21.80 (5.11)	24.21 (5.40)
α	.79	.82	.67	.76	.85

*p < 0.05

**p < 0.01

Task engagement was measured with the Dundee Stress State Questionnaire (DSSQ; [18]) in the short version [28], translated into Polish [27]. It contains eight items assessing task engagement with 5-point response scales. Example items are "I am/was determined to succeed in the task", "My attention will be/was directed towards the task", "I feel/felt energetic". The internal consistency of the Polish version is high (task engagement α = 0.80). The DSSQ was administered twice: just before and immediately after Raven's test.

Trait Anger was assessed with the scale from the State Trait Anger Expression Inventory-2 [29] in a Polish adaptation [30]. It consists of ten items (e.g., “I have a fiery temper”) with a 4-point type response format (from 1 –Almost Never to 4 –Almost Always).

Agreeableness was measured with the same tool as in study 3.

Intelligence was assessed with Raven’s Advanced Progressive Matrices Test (APM; [31]). The test consists of 36 items that include a three-by-three matrix of figural patterns which is missing the bottom-left pattern. Additionally, there are eight responses which potentially match a missing part. The score in the Raven’s test is the total number of correct responses. APM is regarded as a good marker of fluid ability.

#### Results

Table 5 presents the correlations of all the variables. We found that anger was negatively associated with agreeableness, task engagement measured after intelligence test, and Raven's test. Agreeableness correlated positively with task engagement, but only in the pre-test situation. Finally, the high level of the second task engagement measurement correlated positively with Raven's test.

**Fig 4:**
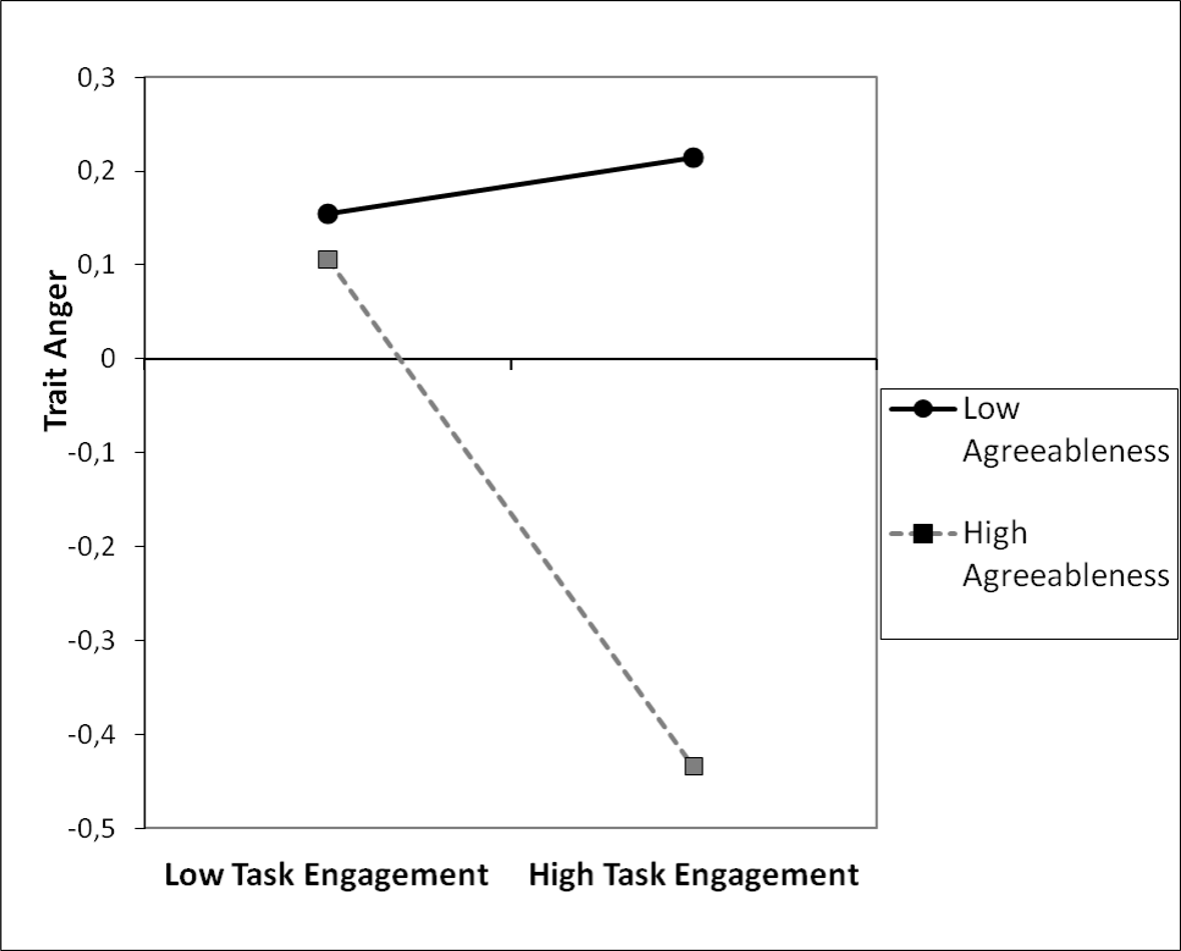
Trait anger as a function of agreeableness and task engagement, study 4.

Subsequently, the interaction between agreeableness and task engagement in predicting trait anger was tested. Two separate analyses were conducted for each measurement of task engagement. However, the interaction was significant only in the post-task situation (see Table 6). This is consistent with previous findings and suggestion that the post-task score is most representative of state during task performance [28]. Furthermore, we examined the simple slope of task engagement among individuals high in agreeableness (1 *SD* above the mean) and low in agreeableness (1 *SD* below the mean). Fig 4 shows graphically that at high level of agreeableness, the association between task engagement and anger was more pronounced and negative (β = -0.26, *t* (185) = -2.67, *p* = .008), while for individuals low in agreeableness this relationship was not significant (β = 0.03, *t* (185) = 0.32, *p* = .750). The Johnson-Neyman regions of significance test revealed that task engagement was negatively associated with anger among individuals who scored + 0.20 of a standard deviation or higher on agreeableness.

**Table 6:** Regression models with agreeableness, task engagement (pre and post-task), and agreeableness x task engagement interaction as predictors and anger as a dependent variable.

Model 1	β	p	Model 2	β	p
Agree	-.17	.020	Agree	-.17	.016
TE pre-task	-.09	.208	TE post-task	-.11	.111
Agree x TE pre-task	-.05	.465	Agree x TE post-task	-.14	.049

*Note* TE–task engagement

**Fig 5:**
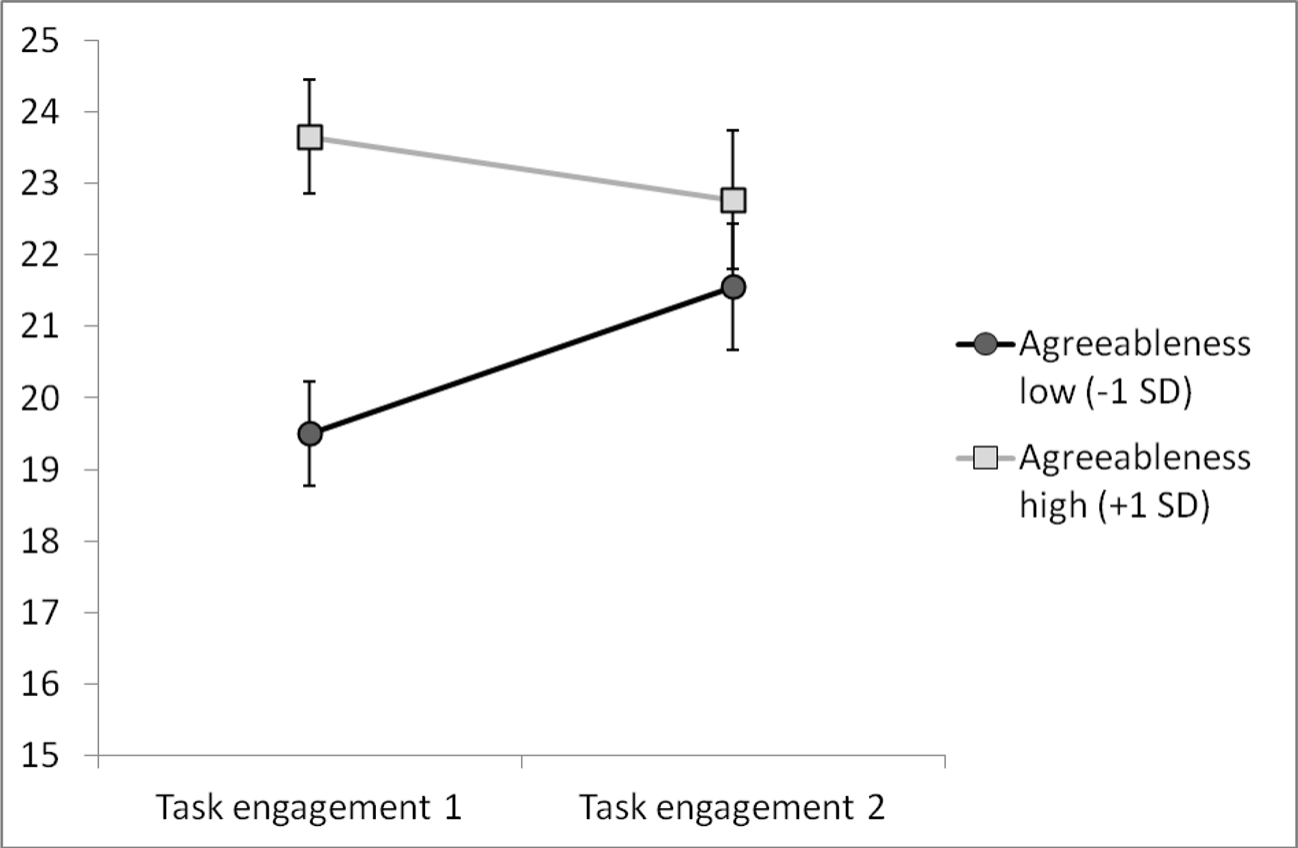
Changes in task engagement between participants scoring low and high on agreeableness.

In order to test how the level of task engagement changed while taking an intelligence test, repeated measures ANOVA (with two measurements of task engagement as the within subjects factor) was performed. The analysis revealed that for all participants task engagement increased after the test (*F*(1, 187) = 7.74; *p* = 0.006; η^2^ = 0.04, before test *M* = 20.92, SE = 0.31; after test *M* = 21.87, *SE* = 0.36). Furthermore, we decided to examine the role of agreeableness in the changes in task engagement. Thus, we selected participants scoring low and high on the agreeableness scale (1 *SD* below and 1 *SD* above the mean, respectively). ANOVA with the two task engagement measurements (within subjects factor) and two levels of agreeableness (between subjects factor) revealed a significant interaction effect (*F*(1, 56) = 5.85, *p* = 0.020, η^2^ = 0.10). Further analysis of the interaction indicated that individuals with low and high agreeableness differed significantly in the first measurement (*F*(1,56) = 14.94, *p* = 0.000, η^2^ = 0.21; *M* = 19.50, *SE* = 0.72 for low agreeableness, *M* = 23.65, SE = 0.80 for high agreeableness), while in the second measurement there was no difference (*F*(1,56) = 0.85, *p* = 0.362, η^2^ = 0.02, *M* = 21.56, *SE* = 0.88 for low agreeableness, *M* = 22.77, *SE* = 0.97 for high agreeableness). Moreover, the level of task engagement changed (increased) significantly only among participants with low agreeableness (*F*(1, 31) = 9.77, *p* = 0.004, η^2^ = 0.24; see Fig 5).

**Fig 6:**
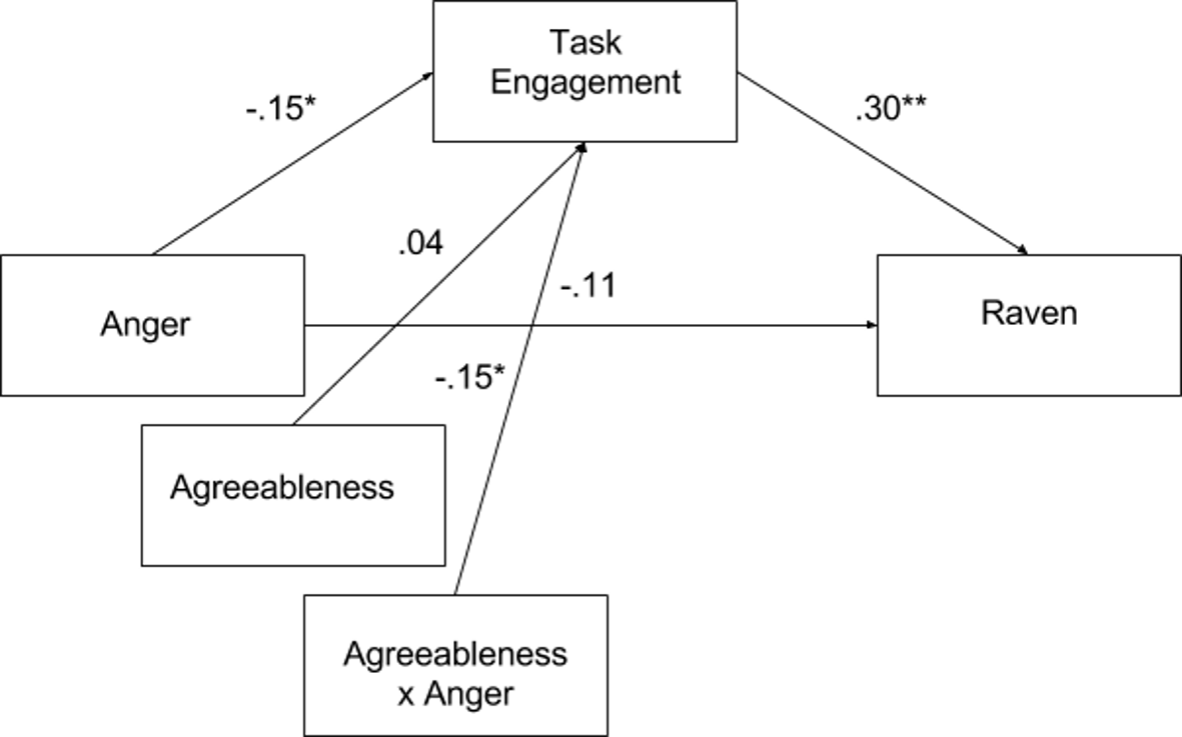
Diagram of the moderated mediation model. *p < 0.05, **p < 0.01.

Finally, we examined the relationship between variables in one model. Prior studies have shown that states related to intelligence tests mediate the relationship between personality traits and performance on that test (e.g. [26], [27]). Therefore, we decided to examine whether task engagement mediates the relationship between trait anger and Raven's score. Moreover, our analyses presented above suggested that agreeableness moderates the relationship between task engagement and anger. Thus, we included agreeableness as a potential moderator of the mediation effect. The post-task score for task engagement was used since it was suggested this is most representative of state during task performance [28]. In order to illustrate these effects in a model that would reflect the dynamics of obtained associations, we used the PROCESS macro for SPSS (model 7; [32]), which tests for moderated mediation by calculating confidence intervals using bootstrapping (2,000 bootstrapped samples in the present analysis; see Fig 6).

The analysis has shown a significant effect of moderated mediation (-.043, 95% CI [-.09; -.01]. Subsequent analysis revealed that task engagement mediated the relationship between anger and the intelligence test only at the high (+ 1 SD) level of agreeableness (indirect effect -.08, 95% CI [-.17, -.02]), while at the medium (indirect effect -.04, 95% CI [-.10, .01]) and low (- 1 SD) levels of agreeableness (indirect effect .01, 95% CI [-.06, .07]) mediation was not significant.

#### Discussion

The last study once again revealed that trait anger is predicted by an interaction of agreeableness and EA-related construct. This time, however, it was found that task engagement after taking an intelligence test was negatively associated with trait anger at a high level of agreeableness, whereas at low agreeableness there was no significant correlation between anger and task engagement. Moreover, it was also found that the baseline level of task engagement among disagreeable individuals was low in comparison to agreeable individuals, and increased after cognitive performance. Finally, the moderated mediation model indicated that agreeableness may act as a moderator of the task engagement mediation between anger and the intelligence test. Specifically, low agreeableness counteracted the negative effect of anger on task engagement.

## General discussion

The current studies examined the interaction between agreeableness, EA, and EA related constructs, in predicting trait anger. In four studies, a prediction was tested that trait anger is a mixture of low agreeableness and high EA. Studies 1 and 2 confirmed the hypothesis, extending past work [4] by showing a direct association of anger and EA (study 1) which is relatively stable over time (study 2). Subsequent studies indicated that the observed effect generalizes to EA related constructs, i.e., positive affect and, to some extent, task engagement.

Among the Big Five personality traits, agreeableness and neuroticism are typically linked with anger and aggression [2]. It has been suggested that these traits might play distinct roles in the self-regulation of anger. Ode, Wilkowski, and Robinson [33] have argued that high neuroticism facilitates emotional aspects of anger, while agreeableness is more related to cognitive mechanisms of anger regulation. Empirical data imply that agreeable and disagreeable individuals might differ in their beliefs about relationships and the content of the social knowledge in long term memory [16]. For instance, disagreeable people are more likely to believe in the hostility and malevolence of other people [34]. Moreover, investigations indicate that dispositional anger predicts the accessibility of hostile thoughts, but only at the low level of agreeableness, while at the high level of agreeableness this relationship is non-significant [35]. Disagreeableness seems to represent cognitive aspects related to high trait anger, i.e., increased hostility and hostile attribution bias [7]. The current studies suggest that this attitude might be a condition, or a trigger, of energy mobilization for angry individuals.

The question arises about the mechanism underlying the moderating role of agreeableness in the anger-energy relation. Some researchers suggest that cognitive reappraisal characteristic for agreeable individuals might be engaged in the regulation of negative emotionality, especially related to anger and aggression [35]. This idea is based on the empirical findings showing that agreeable individuals expand more effort to control negative emotions produced by negatively valenced stimuli emotions relative to individuals lower in agreeableness [36], [37]. This increased effort seems to rely on cognitive processing. Meier, Robinson and Wilkowski [38] argued that agreeable individuals tend to self-activate prosocial thoughts in response to aggression-related primes; for instance, they are faster to categorize prosocial words (e.g., help, love) primed with hostile stimuli. The current studies (1–3) suggest a disconnect between energy and anger among those high in agreeableness. Taken this result together with other findings related to cognitive regulation of anger, one may wonder whether high agreeableness inhibits energy/anger relation by weakening the impact of hostile thoughts. In contrast, lack of such cognitive strategies at a low level of agreeableness may serve to facilitate the energy-anger relation. It has to be acknowledged, however, that this is a speculative interpretation and should be carefully examined in future studies.

The present investigation extends also our knowledge on the nature of PA measured with the PANAS. Study 3 revealed that positive affect exhibits similar effects to EA. Previously, Harmon-Jones et al. [12] postulated that anger is positively associated with PA, because the latter taps approach motivation. As was noticed in the introduction, it is it difficult to clearly distinguish between PA and EA, especially when they are measured with the means of self-report methods. Moreover, some researchers consider EA as a broad construct that may actually capture various phenomena. For instance, Thayer [10] conceptualized EA as a 'general appetitive or action system that also has an important signal function for self-monitoring and decision making. It is most readily identifiable through feelings that range from energy, vigor and liveliness to states of fatigue and tiredness. (p. 134)'. Thus, EA appears to combine biological, motivational and even cognitive aspects of certain state of the organism.

The results obtained in the current research may shed new light on the cognitive correlates of anger. A number of works suggest that trait anger is associated with poorer cognitive functioning. For instance, trait anger is negatively related to intelligence [39], and executive functions [27]. Interesting findings concern also the recruitment of cognitive control resources within hostile situations of individuals with low trait anger [7]. All these findings suggest that high trait anger individuals have difficulties in cognitive performance. The present research shows, however, that the relationship between anger and cognition might be more complex, and perhaps, in some cases, anger may enhance cognitive functioning. Trait anger, considered together with low agreeableness, was consequently linked to high energy. High EA is usually more beneficial to attention, while the relationship between energy and other cognitive functions (e.g. short- long long-term memory) is more ambiguous (e.g. [40] [41]). Humphreys and Revelle [40] observed the facilitative effect of EA on sustained attention, measured through tests such as letter cancellation, vigilance tasks, or simple arithmetic. The authors interpreted these results as suggesting that increases in arousal may make information-processing resources more available. Thus, it would be interesting to see in future studies whether trait anger, coupled with low agreeableness, is positively associated with simple attentional tasks through increased EA. Furthermore, study 4 has shown that high anger and low agreeableness may counteract the negative consequences of anger on task engagement. The latter is a complex construct consisting of motivation, concentration, and EA. Taking the result of study 4 with other studies reported here, one may assume that it is the factors related to energy/fatigue that do not decrease among disagreeable individuals. Further investigations may therefore explore how anger and agreeableness are related to energy consuming tasks, and potentially counteract fatigue in cognitive performance.

The present investigation has several limitations. First, the studies were mostly correlational in nature (studies 1–3), with attempts to capture some dynamics in study 4. It would be valuable, however, to use other procedures, such as the experimental design proposed by Harmon-Jones et al. [12]. These authors examined the association between anger and PA in the situations evoking the subjective experience of anger (e.g. insult). They found that provocation elevates both state anger and PA. It would be interesting to see whether agreeableness moderates the association between state anger and EA, especially in the context of evoked anger. Finally, in the reported studies, EA was assessed only with self-report measures. Although there is evidence showing that EA correlates with physiological indexes of arousal [10], adding biological measures would strength the present conclusions that anger is linked to energy at low level of agreeableness.
